# Evaluating Catheter Ablation Versus Conventional Management for Ventricular Arrhythmias in Arrhythmogenic Right Ventricular Cardiomyopathy: A Five-Year Retrospective Cohort Study

**DOI:** 10.7759/cureus.56709

**Published:** 2024-03-22

**Authors:** Fahad R Khan, Shakeel Ahmed Memon, Wasim Sajjad

**Affiliations:** 1 Cardiology, Lady Reading Hospital, Peshawar, PAK

**Keywords:** conventional therapy, retrospective cohort study, ventricular arrhythmias, catheter ablation, arrhythmogenic right ventricular cardiomyopathy (arvc)

## Abstract

Background

Arrhythmogenic right ventricular cardiomyopathy (ARVC) is a challenging genetic disorder marked by ventricular arrhythmias and sudden cardiac death, particularly in athletes and young adults. Despite its clinical significance, the relative effectiveness and safety of catheter ablation versus conventional management in ARVC are not fully delineated.

Objective

This study evaluates the efficacy and safety of catheter ablation compared to conventional management in reducing ventricular arrhythmias and improving patient outcomes over five years in ARVC patients.

Methods

In a retrospective cohort design at Lady Reading Hospital, Peshawar, we analyzed 120 ARVC patients from January 2018 to December 2023. Patients were divided into two groups: those undergoing catheter ablation and those receiving conventional management. Primary outcomes assessed were recurrence of ventricular arrhythmias, procedural complications, hospitalization duration, and mortality rates. Logistic regression was adjusted for demographics and clinical variables.

Results

Catheter ablation significantly lowered the recurrence of ventricular arrhythmias (20% vs. 55%, p<0.01) and reduced hospital stay duration (4 ± 2 days vs. 7 ± 3 days, p<0.05). A trend toward reduced five-year mortality was observed in the catheter ablation group (5% vs. 15%, p=0.07). Age, New York Heart Association class, and exercise capacity emerged as significant predictors of outcomes.

Conclusions

Catheter ablation outperforms conventional management in reducing the recurrence of ventricular arrhythmias and hospitalization in ARVC patients, with a promising trend toward enhanced survival. These findings advocate for personalized management strategies in ARVC, highlighting the necessity for further research to establish the long-term benefits of catheter ablation.

## Introduction

Arrhythmogenic right ventricular cardiomyopathy (ARVC) represents a unique clinical challenge characterized by the progressive replacement of right ventricular myocardial tissue with fibro-fatty material. This genetic disorder is a notable cause of ventricular arrhythmias and sudden cardiac death, especially in young adults and athletes, making it a focal point of cardiovascular research and clinical intervention strategies [1,2]. Despite considerable advancements in understanding ARVC's genetic underpinnings and clinical manifestations, the pathophysiology and disease progression remain incompletely elucidated, complicating effective management and treatment [3,4].

Current treatment strategies for ARVC range from conservative management, including lifestyle modifications and pharmacotherapy, to more invasive approaches such as the implantation of cardioverter-defibrillators and catheter ablation. The advent of catheter ablation as a treatment for controlling ventricular arrhythmias has been a significant development, offering a new avenue for reducing arrhythmia recurrence and improving patient outcomes. However, comprehensive evaluations comparing the efficacy and safety of catheter ablation to conventional management methods in ARVC patients are notably lacking, creating a gap in our collective knowledge [5-7].

This gap is not trivial, considering the potential of catheter ablation to offer substantial therapeutic benefits. With the evolving landscape of ARVC diagnosis, treatment, and risk stratification, there is a pressing need for updated comparative analyses of these treatment modalities. Such analyses are crucial for informing clinical decision-making and optimizing patient care strategies [8,9]. Moreover, the varying effectiveness of these treatments across different patient demographics and clinical presentations highlights the need for personalized treatment approaches, further underscoring the importance of this study.

In response to these challenges, our study aims to bridge the existing knowledge gap by providing a comprehensive analysis of the comparative efficacy and safety of catheter ablation versus conventional management in ARVC patients. Through a retrospective analysis of clinical data spanning five years, this research endeavors to illuminate optimal care strategies for this difficult-to-manage patient population, potentially guiding future clinical practices and therapeutic developments. This research is not only timely but also essential for advancing our understanding and management of ARVC, promising to contribute valuable insights to the field of cardiovascular medicine.

## Materials and methods

Study design and setting

This study utilized a retrospective cohort design, conducted at Lady Reading Hospital, Peshawar, which is a tertiary care hospital, covering a period from January 2018 to December 2023. Its primary objective was to compare the effectiveness and safety of catheter ablation versus conventional management in patients diagnosed with ARVC. A retrospective cohort study, such as this, involves analyzing previously collected data from patient records to assess and compare outcomes over time. Specifically, we reviewed medical records to identify ARVC patients, categorizing them into two groups based on their treatment type: catheter ablation or conventional management. This design allowed us to utilize existing clinical data to track the recurrence of ventricular arrhythmias, document procedural complications, and measure hospitalization durations and mortality rates retrospectively. By comparing these outcomes between the two treatment groups, the study provides insights into the relative effectiveness and safety of catheter ablation compared to more traditional management approaches in a real-world clinical setting.

Participant Selection

A total of 120 patients diagnosed with ARVC were included, divided equally into two cohorts: the catheter ablation group and the control group, which received conventional management.

Inclusion Criteria

The inclusion criteria were based on a thorough clinical assessment, ensuring a robust comparison between the two treatment modalities. Efforts to minimize selection bias included standardized retrospective review procedures and criteria for inclusion that were strictly adhered to across all cases.

Exclusion Criteria

Patients were excluded if they had other forms of cardiomyopathy, significant coronary artery disease, previous heart surgery, or incomplete medical records. We also excluded patients who declined participation in the study or had contraindications to catheter ablation.

Conventional management for ARVC patients in this study encompasses a combination of lifestyle modification advice, pharmacological treatment (including beta-blockers, angiotensin-converting enzyme inhibitors, or antiarrhythmic drugs), and, when necessary, the use of implantable cardioverter defibrillators (ICDs) for arrhythmia prevention. This approach follows current clinical guidelines and is tailored to each patient's disease severity and symptoms.

Catheter Ablation Techniques

Catheter ablation was performed using state-of-the-art 3D electroanatomic mapping systems, primarily utilizing the CARTO system for real-time visualization and mapping of the heart's electrical activity. This facilitated the precise identification and targeting of arrhythmogenic substrates within the right ventricle. The ablation strategy focused on substrate modification, targeting areas exhibiting abnormal electrograms indicative of arrhythmogenic tissue, including fractionated signals and late potentials. The approach was tailored to each patient, with most undergoing an endocardial approach and a combined endo-epicardial approach employed in select cases where epicardial arrhythmogenic foci were suspected. Ablation endpoints included the elimination of all inducible ventricular tachycardias with programmed stimulation and the abolition of abnormal electrograms within the targeted areas.

Data Compilation

The data were compiled by a dedicated team comprising clinical researchers and data analysts trained in ARVC management and catheter ablation procedures. This team systematically reviewed patient medical records to extract relevant demographic details, diagnostic criteria for ARVC, characteristics of ventricular arrhythmias, details of catheter ablation procedures, and comprehensive follow-up outcomes. To ensure the accuracy and reliability of the data, double data entry was employed for all information extracted from the medical records. Regular data audits were conducted to identify and rectify any discrepancies or inconsistencies, ensuring the integrity of the compiled dataset. Key variables examined included age, gender, New York Heart Association (NYHA) functional class, type of ventricular arrhythmia, and prior ICD placements.

Outcome Measures

Primary outcomes of interest were the recurrence rate of ventricular arrhythmias, incidence of procedure-related complications, duration of hospital stays, and mortality rates over the study period. The selection of these outcomes was intended to provide a comprehensive view of the efficacy and safety of catheter ablation compared to conventional management.

Statistical analysis

Our statistical analysis utilized logistic regression to explore potential associations between patient characteristics (age, gender, NYHA functional class, type of ventricular arrhythmia, prior ICD placement) and primary outcomes (arrhythmia recurrence, procedural complications, hospitalization duration, mortality rates). The selection of these variables was informed by their clinical relevance and potential impact on treatment outcomes. Assumptions underlying the logistic regression analysis, including the linearity of the logit for continuous variables, the absence of multicollinearity, and the independence of observations, were carefully verified. Sensitivity analyses were conducted to assess the robustness of our findings across different patient subgroups and to account for any variability in follow-up durations.

Ethical Considerations

The study received approval from the Institutional Review Board of Lady Reading Hospital, Peshawar (approval number: 107/LRH/MTI, approval date: December 25, 2017), ensuring adherence to ethical standards. Informed consent was obtained from all participants, in alignment with the principles outlined in the Declaration of Helsinki. Given its retrospective nature, the requirement for informed consent was waived. This decision was made in compliance with ethical guidelines, prioritizing patient confidentiality and data integrity. Measures were implemented to anonymize patient data and maintain the confidentiality of patient records throughout the study.

Bias Mitigation and Data Integrity

To address potential retrospective biases, several strategies were employed, including rigorous validation of ARVC diagnoses using established criteria and ensuring uniformity in data collection procedures. Data integrity was maintained through systematic data review and validation processes, overseen by a dedicated team trained in handling medical records for research purposes.

## Results

The study analyzed data from 120 patients diagnosed with ARVC, equally divided into the catheter ablation group and the control group (n=60 (50%) each). Baseline demographic and clinical characteristics were similar across both groups, facilitating a balanced comparison of treatment outcomes, as detailed in Table 1.

**Table 1:** Baseline demographic and clinical characteristics NYHA: New York Heart Association, VT: ventricular tachycardia, VF: ventricular fibrillation, ICD: implantable cardioverter defibrillator

Characteristic	Catheter Ablation Group	Control Group
Age (years)	45 ± 10	47 ± 11
Male	35 (58%)	30 (50%)
NYHA Class I	10 (17%)	15 (25%)
NYHA Class II	25 (42%)	20 (33%)
NYHA Class III	20 (33%)	20 (33%)
NYHA Class IV	5 (8%)	5 (8%)
VT	45 (75%)	50 (83%)
VF	15 (25%)	10 (17%)
Prior ICD Placement	30 (50%)	25 (42%)

Significant differences were observed between the groups. The catheter ablation group demonstrated a notably lower recurrence rate of ventricular arrhythmias and a reduced average hospitalization duration compared to the control group. The trend toward improved five-year mortality rates in the catheter ablation group, although not statistically significant, suggests a potential benefit warranting further investigation, as shown in Table 2.

**Table 2:** Outcome measures post-treatment N/A: not applicable due to the nature of conventional management not involving procedural interventions

Outcome Measure	Catheter Ablation Group	Control Group	P-value
Ventricular arrhythmia recurrence	12 (20%)	33 (55%)	<0.01
Procedure-related complications	6 (10%)	N/A	N/A
Hospitalization duration, days (mean ± SD)	4 ± 2	7 ± 3	<0.05
Mortality at 5 years	3 (5%)	9 (15%)	0.07

Logistic regression analysis was conducted to identify predictors of arrhythmia recurrence and quality of life improvements. It highlighted both the statistical and clinical implications of age, NYHA class, and exercise capacity on the treatment outcomes for ARVC, with each factor carrying distinct implications for clinical practice. The finding that each additional year of age was associated with decreased odds of arrhythmia recurrence (OR: 0.92; 95% CI: 0.87-0.97) suggests a nuanced relationship between age and ARVC progression, pointing toward potentially more aggressive disease management or monitoring in younger patients. Similarly, the NYHA class's significant impact (OR: 0.45; 95% CI: 0.22-0.93) emphasizes heart failure's role in ARVC's clinical course, indicating that higher NYHA classes, paradoxically associated with lower arrhythmia recurrence, may reflect a complex interplay between heart failure management and arrhythmia control strategies. The effect of exercise capacity on quality of life improvements (OR: 1.10; 95% CI: 1.02-1.18) further underscores the importance of physical conditioning and its potential role in patient management. These insights not only demonstrate the factors' statistical significance but also their practical and clinical importance, guiding clinicians toward more personalized, nuanced care strategies for ARVC patients, as illustrated in Table 3.

**Table 3:** Logistic regression analysis: predictors of key outcomes OR: odds ratio, CI: confidence interval

Predictor	Outcome	OR	95% CI	P-value
Age	Arrhythmia recurrence	0.92	0.87-0.97	<0.05
NYHA class	Arrhythmia recurrence	0.45	0.22-0.93	<0.05
Exercise capacity	Quality of life improvement	1.10	1.02-1.18	<0.05

Building upon the foundation of observed treatment outcomes, Table 4 presents a multiple logistic regression analysis. This analysis further elucidates the complex interplay of factors influencing key outcomes, providing a comprehensive view of the predictors associated with arrhythmia recurrence, hospitalization duration, and improvements in quality of life and functional status. Such insights are instrumental in refining ARVC management strategies.

**Table 4:** Multiple logistic regression analysis: comprehensive outcome predictors aOR reflects the influence of multiple factors on the outcomes aOR: adjusted odds ratio, CI: confidence interval

Outcome	aOR	95% CI	P-value
Arrhythmia recurrence	0.33	0.12-0.89	<0.05
Hospitalization duration	0.58	0.35-0.96	<0.05
Quality of life improvement	2.00	1.50-2.67	<0.01
Functional status improvement	1.75	1.10-2.80	<0.05

Our findings reveal statistically significant differences between groups in the recurrence rate of ventricular arrhythmias and hospitalization duration, underscoring the efficacy of catheter ablation in reducing arrhythmic events and shortening hospital stays. Although the observed trend toward improved five-year mortality rates in the catheter ablation group did not reach statistical significance (p=0.07), its clinical relevance cannot be overlooked. This trend suggests a potential for life-extending benefits from catheter ablation, warranting further investigation through larger, prospective studies.

## Discussion

Our five-year-long retrospective cohort study offers valuable insights into the comparative efficacy and safety of catheter ablation versus conventional management in patients diagnosed with ARVC. This is consistent with prior research indicating the potential benefits of catheter ablation in managing ventricular arrhythmias [10,6]. Our findings demonstrate a significant reduction in the recurrence of ventricular arrhythmias in the catheter ablation group compared to the control group. This reduction underscores the importance of a targeted therapeutic approach in ARVC management, aligning with the evolving paradigm of precision medicine in cardiology [2,3].

The safety profile of catheter ablation, as observed in our study, with a low incidence of procedure-related complications, provides a reassuring confirmation of the procedure’s relative safety when conducted in a specialized setting. This is particularly relevant given the ongoing debate about the risk-benefit ratio of invasive strategies in ARVC treatment [5,11]. Our findings contribute to the growing body of evidence suggesting that, with appropriate patient selection and procedural expertise, catheter ablation can be a safe and effective management strategy for ARVC.

Interestingly, our study noted a non-significant trend toward reduced mortality in the catheter ablation group. While not statistically significant, this trend invites further investigation into the potential for catheter ablation to confer long-term survival benefits, a question that has been previously raised but remains unresolved in the literature [7,8]. The complexity of ARVC’s progression and the multifactorial nature of arrhythmic risk underscore the need for long-term, prospective studies to fully elucidate the impact of catheter ablation on survival.

The reduced hospitalization duration observed in our catheter ablation group not only has implications for patient quality of life but also represents a potential reduction in healthcare resource utilization, a significant consideration in the current era of cost-conscious medical care [9,12]. This aspect of our findings highlights the importance of integrating clinical efficacy with economic considerations in the management of chronic conditions like ARVC.

Our study’s unique contribution lies in its comprehensive analysis over a significant timeframe, providing a robust dataset for evaluating the long-term outcomes of catheter ablation versus conventional management in ARVC. By filling a critical gap in the literature, our research supports a more nuanced understanding of ARVC management strategies and their implications for patient outcomes.

Limitations

This study, notwithstanding its contributions, is not without limitations. The retrospective design and the single-center scope may restrict the generalizability of the findings. Prospective multicenter studies are warranted to validate these results and explore the nuances of catheter ablation’s impact on long-term survival rates and quality of life in ARVC patients.

## Conclusions

Our study substantiates the role of catheter ablation as a superior management strategy for ARVC, marked by its effectiveness in reducing arrhythmia recurrence and its favorable safety profile. It heralds a shift toward more individualized treatment paradigms, accentuating the need for ongoing research and innovation in cardiovascular medicine.

## References

[1] Welkie R (2023). Understanding arrhythmogenic right ventricular cardiomyopathy. JAAPA.

[2] Odak M, Douedi S, Mararenko A (2022). Arrhythmogenic right ventricular cardiomyopathy: the role of genetics in diagnosis, management, and screening. Cardiol Res.

[3] Dolkar T, Nway N, Hamad AM, Jain H, Dufresne A (2022). Arrhythmogenic right ventricular cardiomyopathy. Cureus.

[4] Alblaihed L, Kositz C, Brady WJ, Al-Salamah T, Mattu A (2023). Diagnosis and management of arrhythmogenic right ventricular cardiomyopathy. Am J Emerg Med.

[5] Al-Aidarous S, Protonotarios A, Elliott PM, Lambiase PD (2024). Management of arrhythmogenic right ventricular cardiomyopathy. Heart.

[6] Caixal G, Waight M, Pinto A, O Neill C, Grimster A, Li A, Saba M (2023). Safety and efficacy of bipolar radiofrequency catheter ablation in ventricular arrhythmias of mural vs non-mural origin. Europace.

[7] Huseynli EG, Sapelnikov OV, Amanatova VA (2023). Efficacy and safety of nonfluoroscopic approach during catheter ablation of ventricular tachycardia (Article in Russian). Kardiologiia.

[8] Cadrin-Tourigny J, Bosman LP, James CA (2022). Sudden cardiac death risk prediction in arrhythmogenic right ventricular cardiomyopathy: a practical approach to navigating the challenges of prediction models. Eur Heart J.

[9] Al-Sinan A, Swampillai J, DeBoard Z, Ho C, Stiles M (2023). Hybrid epicardial ventricular ablation for arrhythmogenic right ventricular cardiomyopathy. Heart Lung Circ.

[10] Kotake Y, Huang K, Bennett R (2023). Efficacy and safety of catheter ablation as first-line therapy for the management of ventricular tachycardia. J Interv Card Electrophysiol.

[11] Romero J, Díaz JC, Cerna L (2022). Abstract 15689: efficacy and safety of non-fluoroscopic radiofrequency ablation of ventricular arrhythmias originating in the sinuses of Valsalva. Circ.

[12] Coteli C, Zekeriyayev S, Ates AH, Yorgun H, Aytemir K (2023). Long term results of catheter ablation in patients presented with ventricular storm. Europace.

